# Comparison of endoscopic retrograde cholangiopancreatography outcomes between cap-fitted forward and side viewing endoscopes in patients with Billroth II anastomosis

**DOI:** 10.1186/s12876-023-02701-x

**Published:** 2023-04-06

**Authors:** Sung Bum Kim, Kook Hyun Kim, Tae Nyeun Kim

**Affiliations:** 1 Department of Internal Medicine, Yeungnam University College of Medicine, Daegu, Korea; 2 Division of Gastroenterology and Hepatology, Department of Internal Medicine, Yeungnam University College of Medicine, 170 Hyeonchung-ro, Nam-gu, Daegu, 42415 Korea

**Keywords:** Subtotal gastrectomy, Billroth II anatomy, ERCP, Forward viewing endoscope, Side viewing endoscope

## Abstract

**Background:**

There have been no previous studies that directly compared outcomes between cap-fitted forward-viewing and side viewing endoscopes (SE). This study aimed to compare the technical success rate and occurrence of adverse events between the side viewing and cap-fitted forward-viewing endoscope (CE) groups among patients with Billroth II anatomy who underwent ERCP.

**Methods:**

The medical records of patients with a previous history of subtotal gastrectomy using Billroth II reconstruction who underwent ERCP at Yeungnam University Hospital between January 2004 and December 2020 were reviewed retrospectively. The patients were divided into CE and SE group. Propensity score matching analysis was performed to minimize selection bias.

**Results:**

Propensity score matching resulted in 55 matched pairs for further analysis. Patients’ characteristics were comparable in the matched cohorts. Final success rate of selective bile duct cannulation was not significantly different between the SE and CE groups (98.2% vs. 94.5%, p = 0.308). The complete CBD stone removal rate in CBD stone and successful biliary drainage rate in malignant biliary obstruction were not significantly different between the two groups. The rate of total ERCP-related adverse events was higher in the CE group than in the SE group, but the difference was not statistically significant (10.9% vs. 7.3%, p = 0.507). Among adverse events, the rate of post-ERCP pancreatitis showed higher tendency in the CE group than in the SE group (10.9% vs. 5.5%, p = 0.297).

**Conclusion:**

In conclusion, CE seems to be equally effective as SE for ERCP in patients with Billroth II anatomy. However, attention should be paid to development of post ERCP complications, especially pancreatitis, when performed by CE.

## Introduction

Endoscopic retrograde cholangiopancreatography (ERCP) is an essential tool for the diagnosis and treatment of pancreatobiliary diseases and is mainly used for therapeutic purposes because of the risk of adverse events associated with ERCP [1]. The rate of procedural success and adverse events associated with ERCP might be different between patients with normal anatomy and those with surgically altered anatomy. ERCP in patients with surgically altered anatomy requires knowledge of the surgically altered anatomy, use of an adequate type of endoscope, preparation of accessories that fit the endoscope, and the skills of the endoscopist [2, 3].

Frequently encountered surgically altered anatomies in ERCP include anatomies of patients who had undergone Billroth I surgery, Billroth II surgery, Roux-en-Y gastrectomy, total gastrectomy with Roux-en-Y esophagojejunostomy, Whipple surgery, and pylorus-preserving pancreaticoduodenectomy. Among the surgically altered anatomies encountered in ERCP, Billroth II is one of the most frequently encountered surgically altered anatomy in Korea due to the high prevalence of gastric cancer. When performing ERCP for patients with Billroth II anatomy, there are some hurdles to overcome: intubation of the afferent loop, which could be hampered by the angulation and length of the afferent loop; cannulation of the inverted major duodenal papilla; and performing sphincterotomy in the reverse direction [4]. In normal anatomy, a side-viewing endoscope (SE) is primarily used for ERCP. However, in surgically altered anatomy, ERCP can be performed using various endoscopes, including SE or forward-viewing endoscopes such as conventional esophagogastroduodenoscope, colonoscope, single-balloon, and double-balloon enteroscope [5]. Currently, conventional SE and cap-fitted forward-viewing endoscope (CE) are the most commonly used endoscopes for ERCP in patients with Billroth II anatomy. In previous studies, the successful afferent loop intubation rates in patients with Billroth II anatomy were reported as 68–100% in SE and 91.3–100% in CE [6–10], and the successful bile duct cannulation rates were reported to be 77.8–100% in SE and 92.3–100% in CE [8, 9, 11–14]. However, most previous studies on the outcomes of ERCP in patients with Billroth II anatomy have reported the results of a single endoscope, either CE or SE, and no previous study has directly compared outcomes between CE and SE. Although this was a retrospective study, this is the first study to compare the clinical outcomes of ERCP between SE and CE groups with Billroth II anatomy.

This study aimed to compare the technical success rates and occurrence of adverse events associated with ERCP between the SE and CE groups among patients with Billroth II anatomy.

## Methods

The medical records of patients with a previous history of subtotal gastrectomy using Billroth II reconstruction who underwent ERCP at Yeungnam University Hospital between January 2004 and December 2020 were reviewed retrospectively. The inclusion criteria were as follows: (1) age > 18 years, (2) naïve papilla, (3) biliary diseases that required ERCP, and (5) ERCP using either SE or CE. The exclusion criteria were as follows: (1) previous history of ERCP, (2) Braun anastomosis, and (3) pancreatic disease requiring ERCP. Demographic data (age, sex, height, weight, and comorbidities including hypertension, diabetes mellitus, cerebrovascular accident, and chronic kidney disease), laboratory findings at admission (white blood cell count, c-reactive protein, total bilirubin, aspartate aminotransferase (AST), alanine aminotransferase (ALT), gamma-glutamyl transferase (GGT), alkaline phosphatase (ALP), albumin, and creatinine), ERCP findings (presence of periampullary diverticula and gallbladder stone, performance of endoscopic sphincterotomy (EST), endoscopic papillary balloon dilatation (EPBD), needle knife fistulotomy (NKF), mechanical lithotripsy (ML), endoscopic retrograde pancreatic drainage (ERPD), and biliary stent placement), and adverse events associated with ERCP (pancreatitis, bleeding, perforation, cholangitis, and cholecystitis) were retrospectively reviewed from medical data. All patients provided written informed consent before the procedure. Approval of our institutional review board was obtained for this study (YUMC 2022-02-027). All methods were carried out in accordance with relevant guidelines and regulations.

**Fig. 1:**
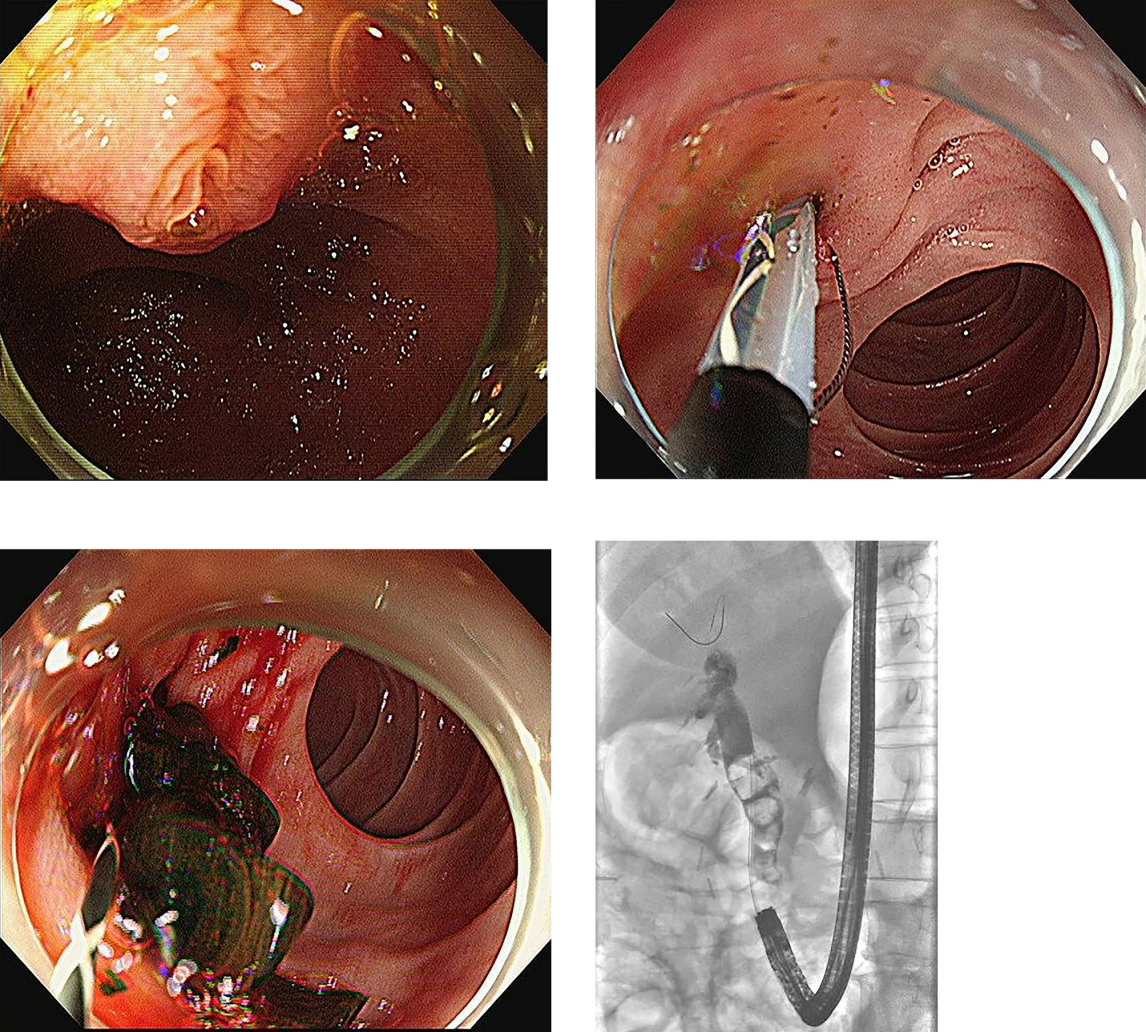
Cap-fitted forward-viewing endoscope. (A) Endoscopic view of naïve papilla (B) Endoscopic sphincterotomy with inverted sphincterotome in reverse direction (C) Removal of choledocholithiasis with balloon (D) Cholangiogram showing dilated bile duct with multiple movable filling defects

**Fig. 2:**
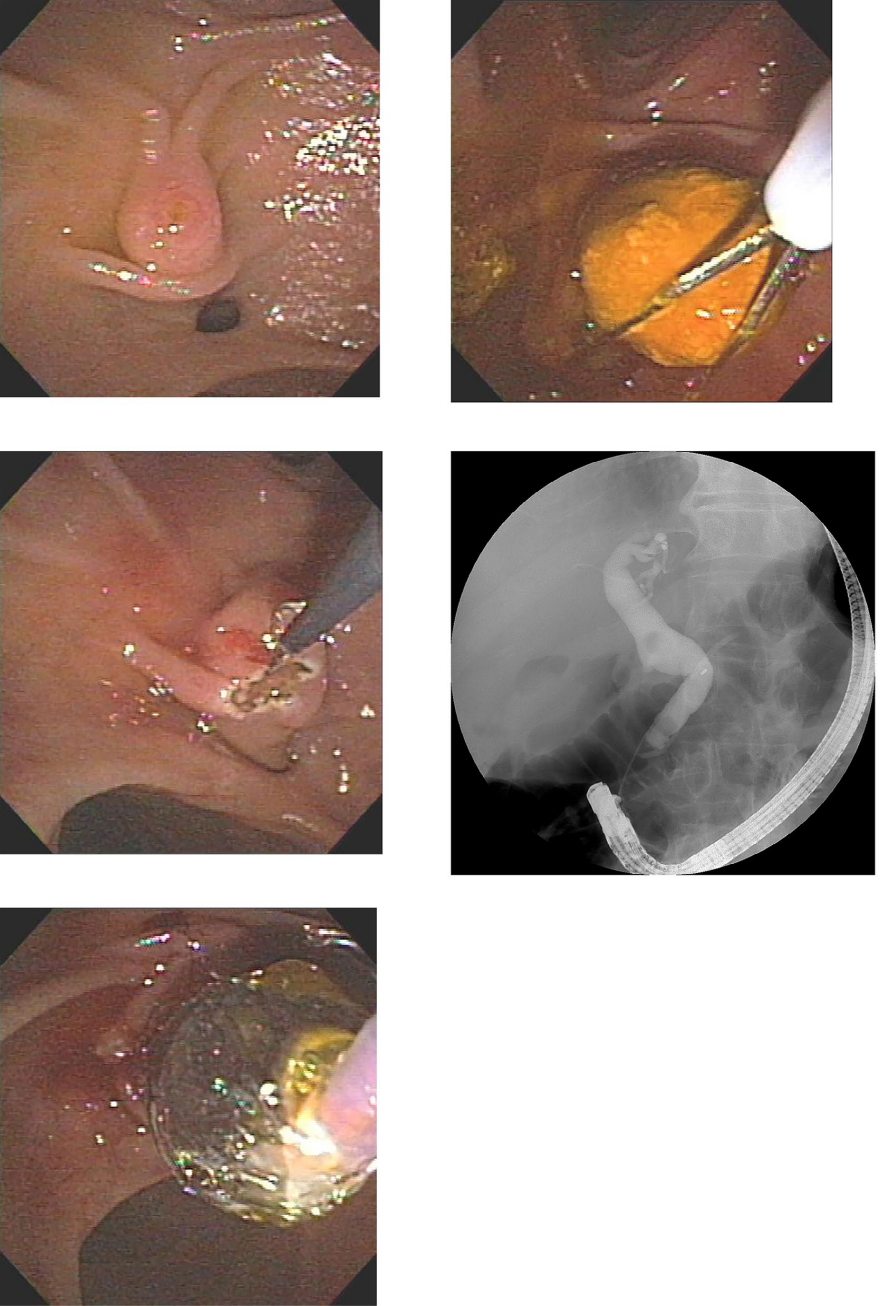
Side viewing endoscope. (A) Endoscopic view of naïve papilla (B) Precutting of naïve papilla with needle knife in reverse direction (C) Endoscopic papillary balloon dilatation (D) Removal of CBD stone with basket (E) Cholangiogram showing dilated bile duct with two movable filling defects

The patients were divided into two groups: CE group and SE group. Type of endoscope used for ERCP in Billroth II anatomy was decided based on the endoscopist’s preference. ERCP was performed by three experienced endoscopists (S.B.K., K.H.K., and T.N.K.); our hospital is a high-volume center that performs more than 600 ERCP cases annually. T.N.K. performed 385 cases of ERCP per year, K.H.K., 441 cases per year, and S.B.K., 338 cases per year during study period. Conscious sedation was performed using midazolam, meperidine, and propofol by the non-anesthesiologist-assisted method. A forward-viewing endoscope (Olympus GIF- Q240/Q260J/Q260/H260; Olympus Optical, Tokyo, Japan) was used in the CE group, and a transparent cap (Disposable distal attachment; Olympus Optical, Tokyo, Japan) was attached to the tip of the endoscope. In the SE group, a side viewing endoscope (Olympus TJF-260 V, JF-260 V; Olympus Optical, Tokyo, Japan) was used. Selective cannulation of the bile duct was initially tried with a catheter (Glo-tip catheter; Cook Medical, Bloomington, IN, USA, Tandem XL; Boston Scientific, Natick, MA, USA) or inverted sphincterotome (Billroth II sphincterotome; Cook Medical, Bloomington, IN, USA) based on endoscopists` decision. Precut sphincterotomy or infundibulotomy was performed using a needle knife (RX needle knife XL; Boston Scientific, Natick, Mass, USA) in cases of difficult biliary cannulation. EPBD was performed using a balloon dilator (CRE Balloon dilator or Titan Balloon dilator; Boston Scientific, Natick, Mass, USA). The balloon size was determined based on the bile duct size and balloon size did not exceeded size of bile duct. The decision to perform NKF, EST, and/or EPBD was based on the endoscopist’s experience. From 2008 to 2014, EST procedures were done with precutting with needle knife under-guidance of plastic stent. From 2014 onwards, EPBD and/or inverted sphincterotome were used for sphincteroplasty based on endoscopists` preference. Mechanical lithotripsy was performed in patients with common bile duct (CBD) stones that were difficult to retrieve with conventional basket and/or balloon. In cases of malignant biliary strictures, stent was inserted using either a plastic stent (Cotton-Leung or Zimmon Biliary stent; Cook Medical, Bloomington, IN, USA) or a self-expandable metal stent (SEMS) (Niti-S; Taewoong medical Inc. Seoul, South Korea). In cases of failed cannulation, ERCP was retried after a few days, based on the endoscopist’s discretion. (Figures 1 and 2).

Technical success included successful selective cannulation of the bile duct, complete removal of CBD stones in patients with CBD stones and insertion of plastic or metal stents and normalization of liver function tests in patients with malignant biliary stricture. Complications of ERCP were defined based on the consensus definition suggested by Cotton et al. [7]. Post-ERCP pancreatitis was defined as a medical condition involving abdominal pain and elevated pancreatic enzymes (amylase or lipase) > three times the upper normal limit. Elevation of serum amylase levels without abdominal pain was defined as post-ERCP hyperamylasemia. Perforation was considered if free air was present in the retroperitoneum or intraperitoneally on the radiograph obtained after ERCP. Post-ERCP bleeding was defined as clinical evidence of bleeding that required intervention or blood transfusion. Patients with newly developed high body temperature and elevated liver function test results after ERCP were diagnosed with cholangitis. Cholecystitis was diagnosed in patients with newly developed right upper quadrant tenderness with thickening of the gallbladder wall on ultrasonography or computed tomography.

### Statistical analysis

Statistical analysis was performed using SPSS (version 25.0, SPSS, Inc., Chicago, IL, USA), and p values of <0.05 was considered statistically significant. Continuous variables were compared using the t-test or Mann–Whitney U test. Categorical variables were analyzed using Fisher’s exact test or χ2 test.

Propensity score matching was performed using a 1:1 nearest neighbor matching algorithm without replacement with distances determined by logistic regression. Propensity score matching was performed based on the following variables: sex, age, performance of EST and EPBD. The optimal matching algorithm was used as a sensitivity analysis.

## Results

### Baseline characteristics

The mean age of the 177 patients was 79.0 ± 9.7 years and male to female ratio was 3.02:1. The most common indication for ERCP was CBD stone in 155 patients (87.6%) followed by malignant biliary stricture in 22 patients (12.4%). A total of 315 ERCP procedures were performed in 177 patients (1.8 procedures per patient). Successful intubation rate of A-loop was not significantly different between CE and SE group (95.8% vs. 93.2%, p = 0.468).

### Comparison between CE and SE group in the unmatched cohort with successful afferent loop intubation

The mean age and male-to-female ratio were not significantly different between the CE and SE groups (78.8 vs. 79.5 years, p = 0.687 and 3.0:1 vs. 2.4:1, p = 0.578, respectively). The mean body mass index (BMI), presence of comorbidities (including hypertension, diabetes mellitus, cerebrovascular accident, and chronic kidney disease), and laboratory test results at admission did not significantly differ between the two groups. CBD stones were the most common reason for ERCP in both groups.

The presence of periampullary diverticula was 15% and 16.4% in the CE and SE groups, respectively (p = 0.823). EST performance was significantly higher in the SE group than in the CE group (45.5% vs. 16.8%, p = < 0.001). EPBD was performed more frequently in the CE group than in the SE group (59.8% vs. 38.2%, p = 0.013). The combination of EST and EPBD did not differ significantly between the CE and SE groups (21.2% vs. 10.9%, p = 0.133). Performance of biliary stent placement was not significantly different between CE and SE groups. Plastic stent was placed in 115 patients (68.5%) and SEMS in 3 patients (1.8%). Among plastic stents, 7 French plastic stent was used in 87 patients and 10 French in 28 patients. Most common length of stent was 5 cm, followed by 7 cm. Mean diameter of largest CBD stone and mean number of CBD stones were not significantly different between two groups.

### Clinical outcomes of ERCP

The success rate of selective bile duct cannulation at the initial session tended to be higher in the SE group than in the CE group without statistical significance (94.5% vs. 87.6%, p = 0.186); the final success rate of selective bile duct cannulation was not significantly different between the SE and CE groups (98.2% vs. 95.6%, p = 0.665). The complete CBD stone removal in CBD stone and successful biliary drainage rates in malignant biliary obstruction were not significantly different between the two groups. The mean number of sessions of ERCP and rate of more than two sessions of ERCP showed a higher tendency in the CE group than in the SE group without statistically significant difference.

### Adverse events of ERCP

The rate of total ERCP-related adverse events was higher in the CE group than in the SE group, but the difference was not statistically significant (15.9% vs. 7.3%, p = 0.147). Among the adverse events, the rate of post-ERCP pancreatitis was higher in the CE group than in the SE group (12.4% vs. 5.5%, p = 0.186). The severity of pancreatitis was mild in most cases, and all patients recovered with conservative treatment. The rate of hyperamylasemia did not differ between the two groups. Perforation developed in one case in each group. Duodenal perforation developed at proximal site of major duodenal papilla in SE group and was managed with endoscopic clipping. Afferent limb perforation developed in CE group and the patient died after surgical primary closure. Infections, including cholangitis and cholecystitis, developed in only two cases in the CE group. The severity of each case of cholangitis and cholecystitis was grade I according to Tokyo guideline 2018 [15, 16]. Mortality developed in two cases of CE group. Causes of mortality were respiratory failure from aspiration pneumonia in one patient and afferent limb perforation in one patient.

**Table 1:** Baseline characteristics of CE and SE group in patients with successful afferent loop intubation

Variables	1:1 Propensity score matching (nearest neighbor matching; n = 110)		
	CE group (n = 55)	SE group (n = 55)	*P-*value
Age (Y)	78.9 ± 11.2	79.5 ± 9.2	0.787
Male:Female	41:14	39:16	0.669
BMI, kg/m^2^	21.5 ± 2.9	20.8 ± 2.7	0.692
Diagnosis			
CBD stones	47 (85.5)	50 (90.9)	0.376
Malignant biliary stricture	8 (14.5)	5 (9.1)	
Comorbidities			
Hypertension	22 (40.0)	16 (29.1)	0.229
Diabetes mellitus	19 (34.5)	14 (25.5)	0.298
CVA	4 (7.3)	3 (5.5)	0.696
Chronic kidney disease	1 (1.8)	0 (0)	0.315
Laboratory findings			
White blood cells, /µL	10,473.3 ± 10,766.4	10,766.4 ± 6,040.6	0.983
C-reactive protein, mg/L	6.2 ± 6.6	6.0 ± 6.0	0.876
Total bilirubin, mg/dL	4.2 ± 4.6	3.0 ± 2.5	0.084
AST, IU/L	521.3 ± 837.5	454.0 ± 1,163.6	0.728
ALT, IU/L	278.5 ± 503.8	218.6 ± 366.8	0.477
ALP, IU/L	578.1 ± 488.4	711.2 ± 485.3	0.155
GGT, IU/L	344.0 ± 269.6	377.4 ± 320.6	0.555
Albumin, g/dL	3.4 ± 0.5	3.6 ± 0.6	0.053
Creatinine, mg/dL	1.0 ± 0.3	0.9 ± 0.3	0.535
Use of antibiotics	44 (84.6)	43 (79.6)	0.503
Use of anti-platelet or coagulant	6 (10.9)	3 (5.5)	0.297
Withdrawal of anti-platelet or coagulant	3 (5.5)	1 (1.8)	0.308

CE, cap fitted forward viewing endoscope; SE, side viewing endoscope; BMI, body mass index; CBD, common bile duct; CVA, cerebrovascular accidents; MI, myocardial infarction; AST, aspartate aminotransferase; ALT, alanine aminotransferase; GGT, gamma-glutamyl transferase; ALP, alkaline phosphatase. Values are presented as mean ± standard deviation or number (%)

### Comparison between CE and SE group in matched cohorts with successful afferent loop intubation

Propensity score matching with 1:1 ratio resulted in 55 matched pairs for further analysis. Both groups were well balanced in all baseline characteristics in the matched cohort by nearest neighbor matching (Table 1). Interventions and findings of ERCP were not significantly different between two groups (Table 2). Successful biliary cannulation rate at initial session showed higher tendency in SE group than CE group without statistical significance (94.5% vs. 83.6%, p = 0.067). The complete CBD stone removal in CBD stone and successful biliary drainage rates in malignant biliary obstruction were not significantly different between the two groups (Table 3).

**Table 2:** Findings and procedures of ERCP in patients with successful afferent loop intubation

Interventions and findings	1:1 Propensity score matching (nearest neighbor matching; n = 110)		
	CE group (n = 55)	SE group (n = 55)	*P-*value
Periampullary diverticulum	6 (10.9)	9 (16.4)	0.405
GB in situ with GB stone	23 (41.8)	23 (41.8)	1.000
EST only	19 (34.5)	25 (45.5)	0.243
EPBD	25 (45.5)	21 (38.2)	0.439
EPLBD	10 (18.2)	12 (21.8)	0.634
EST + EPBD	8 (14.5)	6 (10.9)	0.567
Infundibulotomy	3 (5.5)	2 (1.8)	0.308
Precut sphincterotomy	5 (9.1)	3 (5.5)	0.463
ML	6 (10.9)	5 (9.4)	0.800
ERBD	43 (78.2)	36 (65.5)	0.138
ERPD	6 (10.9)	3 (5.5)	0.297
Guidewire assisted cannulation	49 (89.1)	46 (83.6)	0.405
Unintended pancreatic duct contrast injection	15(29.4)	19(35.2)	0.527
Guidewire in pancreatic duct	8 (14.5)	4 (7.3)	0.221
Largest diameter of CBD stone (mm)	11.3 ± 5.2	11.8 ± 5.0	0.716
Number of CBD stone	2.3 ± 1.9	2.2 ± 1.8	0.833
Largest diameter of CBD (mm)	16.0 ± 4.6	15.8 ± 5.3	0.913
Procedure time (min)	34.5 ± 13.2	29.0 ± 18.0	0.122

CE, cap fitted forward viewing endoscope; SE, side viewing endoscope; NKF, needle knife fistulotomy; EST, endoscopic sphincterotomy; EPBD, endoscopic papillary balloon dilatation; EPLBD, endoscopic papillary large balloon dilatation; ML, mechanical lithotripsy; ERPD, endoscopic retrograde pancreatic drainage; ERBD, endoscopic retrograde biliary drainage; CBD, common bile duct. Values are presented as number (%) or mean ± standard deviation

**Table 3:** Clinical outcomes of ERCP in patients with successful afferent loop intubation

Outcomes	1:1 Propensity score matching (nearest neighbor matching; n = 110)		
	CE group (n = 55)	SE group (n = 55)	*P-*value
Technical success			
Cannulation of bile duct at 1st session	46 (83.6)	52 (94.5)	0.067
Cannulation of the bile duct, final	52 (94.5)	54 (98.2)	0.308
Complete CBD stone removal	42/47 (89.4)	48 / 50 (96.0)	0.207
Successful ERBD in pancreatobiliary malignancy	8/8 (100)	5/5(100)	
Mean sessions of ERCP	1.9 ± 1.2	1.7 ± 1.1	0.414
≥ 2 ERCP sessions	30 (54.5)	25 (45.5)	0.340

CE, cap fitted forward viewing endoscope; SE, side viewing endoscope; CBD, common bile duct; ERBD, endoscopic papillary balloon dilatation; ERCP, endoscopic retrograde cholangiopancreatography. Values are presented as mean ± standard deviation or number (%)

**Table 4:** Post-ERCP adverse events in patients with successful afferent loop intubation

Variables, n(%)	1:1 Propensity score matching (nearest neighbor matching; n = 110)		
	CE group (n = 55)	SE group (n = 55)	*P-*value
Adverse events, total	6 (10.9)	4 (7.3)	0.507
Pancreatitis	6 (10.9)	3 (5.5)	0.297
Mild	5 (9.1)	3 (5.5)	
Moderate	1 (1.1)	0 (0)	
Bleeding	0 (0)	0 (0)	1.000
Perforation	0 (0)	1 (1.8)	0.315
Cholecystitis or cholangitis	0(0)	0 (0)	1.000
Asymptomatic hyperamylasemia	13 (23.6)	15 (27.3)	0.662
Mortality	0 (0)	0 (0)	1.000

CE, cap fitted forward viewing endoscope; SE, side viewing endoscope. Values are presented as number (%)

Rate of post ERCP pancreatitis showed higher tendency in CE group than SE group (10.9% vs. 5.5%, p = 0.297) (Table 4).

## Discussion

Successful afferent loop intubation rate was 95.8% in CE group and 93.2% in SE group, showing comparable result with previous studies [6–10]. Previous studies showed better afferent loop intubation success rate in CE group compared with SE group. Reasons for indifference in successful afferent loop intubation rare might be due to retrospective nature of this study and missed data regarding changes between endoscopes.

In this study, technical success rates of ERCP were comparable between CE and SE in patients with Billroth II anatomy. Successful cannulation of the bile duct was achieved in 95.6% of the CE group and 98.2% of the SE group in unmatched cohort and 94.5% and 98.2% in matched cohort, with results comparable to those of previous reports. In previous studies, successful cannulation rates ranged from 92.3 to 100% with CE [8, 14] and 77.8–100% with SE [11, 13]. Reasons for failed bile duct cannulation were close space and/or angulation between scope and papilla, and instability or looping of scope. To improve success rate of selective biliary cannulation, aligning of biliary axis and catheter by manipulating scope location is important. The complete CBD stone removal rate was 90.0% in the CE group and 96.0% in the SE group in unmatched cohort and 89.4% and 96.0% in matched cohort, and stent insertion was successful in all cases of malignant biliary stricture in both groups. Reasons for incomplete CBD stone removal in both groups were large diameter and high number of CBD stones and stent was placed in case of incomplete CBD stone removal. In previous studies, success rate of ERCP regarding stone removal and stent insertion with CE and SE was between 81.3% and 100% [7, 8, 10, 17], and this study showed results comparable to those of previous reports. Although there has been a study that compared effectiveness of ERCP between SE and forward-viewing endoscope without cap in Billroth II gastrectomy patients [18], no previous study has directly compared outcomes of ERCP performed using CE and of that performed using SE in patients with Billroth II anatomy. Although this was a retrospective study, this is the first study to compare the outcomes of ERCP performed using CE with that of those using SE in patients with Billroth II anatomy.

A study of 75 Billroth II gastrectomy patients who underwent ERCP by SE and forward-viewing endoscope without cap reported equal effectiveness in both endoscopes [16]. A study of 308 patients with Billroth II gastrectomy reported cap-associated gastroscope as predictive factor for technical success of ERCP [17]. A systemic review of 25 studies also reported higher selective cannulation rate in forward viewing endoscope with cap than without cap [18]. When forming ERCP in Billroth II anatomy with forward-viewing endoscope, attachment of cap might increase rate of selective cannulation.

Differences were observed in the performance of ERCP procedures between the CE and SE groups. Infundibulotomy or precut sphincterotomy using a needle knife and EPBD was more frequently performed in the CE group than in the SE group, and EST was more frequently performed in the SE group than in the CE group. These differences in the performance of infundibulotomy, precut sphincterotomy, EPBD, and EST might be due to various factors, including anatomic variation, differences in endoscope type, presence of elevator and working channel diameter. In this study, diverse sphincteroplasty techniques were used over a long study period. For sphincteroplasty in this study, EST using a inverted sphincterotome, EST using a needle knife under-guidance of plastic stent, EPBD alone, and combined EST and EPBD were performed. These diverse techniques used in ERCP might have affected the outcomes and side effects of ERCP in this study. Even after propensity score matching with these factors, outcomes of ERCP showed similar result in matched cohort when compared with unmatched cohort.

The rate of total adverse events related to ERCP was comparable between the CE and SE groups among patients with a Billroth II anatomy. Among the adverse events, asymptomatic hyperamylasemia and post-ERCP pancreatitis were the most common adverse event in this study, showing comparable result with previous study [19]. The rate of post-ERCP pancreatitis tended to be higher in the CE group than in the SE group in this study. A recent meta-analysis of 25 randomized controlled trials showed an increased risk of pancreatitis in overall EPBD compared to EST, and the incidence of PEP was comparable between endoscopic papillary large balloon dilatation plus EST and endoscopic papillary large balloon dilatation alone [20]. A systemic review of ERCP in Billroth II gastrectomy reported that most cases of post-ERCP pancreatitis occurred with EPBD [21]. Therefore, the higher tendency of post-ERCP pancreatitis in the CE group might be due to the significantly higher performance of EPBD in the CE group than in the SE group and a slightly higher failure rate in gaining access to the bile duct at the initial ERCP session in the CE group than in the SE group. As rectal nonsteroidal anti-inflammatory drugs, which has protective effect on development of PEP [22], are unavailable in Korea, rectal nonsteroidal anti-inflammatory drugs were not used for prevention of PEP in this study. Aggressive hydration which preventive effect on PEP was not used in this study [23]. However, the severity of post-ERCP pancreatitis was mild in most cases, and all cases of post-ERCP pancreatitis recovered with conservative treatment. Perforation developed in a single case for each group. In the CE group, type 4 perforation developed after EPBD, and removal of the CBD stone and primary closure of the perforation site in the duodenum was performed surgically. Duodenal perforation developed in the SE group and was managed by endoscopic clipping. Previous systemic review has shown that pancreatitis rate was higher in forward viewing endoscope and perforation rate was higher in side-viewing endoscope [24]. Infections, including cholecystitis and cholangitis, developed only in the CE group. Cholecystitis developed in a patient with gallbladder stones after CBD stone removal and was managed by laparoscopic cholecystectomy. Another case of infection was cholangitis after removal of CBD stone, and it was managed using intravenous antibiotics and conservative treatments.

The advantage of SE for ERCP in patients with Billroth II anatomy is a wider working channel than that of the forward-viewing endoscope, which allows concomitant insertion of various instruments, as in conventional ERCP in patients with normal anatomy, and the use of an elevator. However, intubation of the SE into the afferent loop and advancement of the endoscope tip upto the major duodenal papilla can be technically demanding. Overtube insertion or wire guided intubation might be used to overcome difficulties of afferent loop intubation by SE [25]. The advantage of the forward-viewing endoscope is easier intubation of the afferent loop, reaching the major duodenal papilla, and attachment of the cap to the tip of the forward-viewing endoscope enables regular distance between the mucosal fold and endoscope tip, which assists afferent loop intubation and enhances major duodenal papilla cannulation. However, the smaller working channel of CE, compared to that of SE, presents a technical challenge. In some cases of difficult bile duct cannulation due to acute angulation, the guidewire inserted in the bile duct might serve as a guide in the insertion of a basket or balloon. However, a regular-sized basket or balloon catheter could not be inserted under guidewire guidance in CE. Plastic stents larger than 7 French in diameter could not pass through the working channel of the forward-viewing endoscope. Removal of previously inserted plastic stents larger than 5 French through the working channel is not possible, and withdrawal of the entire endoscope is required for removal of the plastic stent.

This study had some limitations. First, unequal segregation between two groups might have led to selection bias and skewed outcomes. Second, this was a retrospective study, which limits the power of the results. Third, the long study period may have affected the clinical outcomes of this study, as techniques and instruments related to ERCP have evolved over time. The techniques for cannulation, sphincteroplasty, CBD stone removal, and endoscopic biliary and pancreatic drainage were not the same over time. In our center, more cases of ERCP in patients with Billroth II anatomy have been performed with CE than with SE in recent years.

In conclusion, CE seems to be equally effective as SE for ERCP in patients with Billroth II anatomy. However, attention should be paid to development of post ERCP complications, especially pancreatitis when performed by CE.

## Data Availability

The datasets used and/or analysed during the current study available from the corresponding author on reasonable request.
